# Abstracts of the XXVI Congresso Nazionale - Società Italiana di Neonatologia: Venezia, Italy. 7-10 October 2020

**DOI:** 10.1186/s13052-021-01039-y

**Published:** 2021-04-29

## A1: The COVID National Neonatal Registry of the Italian Society of Neonatology (SIN)

### Camilla Gizzi^1^, Lorenza Pugni^2^, Elena Spada^2^, Claudio Bellù^3^

#### ^1^Department of Pediatrics, Ospedale Sandro Pertini, Rome, Italy; ^2^NICU, Fondazione IRCCS Ca’ Granda Ospedale Maggiore Policlinico, Milan, Italy; ^3^Geos Consult S.r.l., Milan, Italy

##### **Correspondence:** Camilla Gizzi (camillagizzi@gmail.com)

**Background:** The COVID National Neonatal Registry aims to collect clinical data of infants born from mothers with SARS-CoV-2 virus infection diagnosed at any time during pregnancy and data from infants with acquired SARS-CoV-2 virus infection within the first month of life.

**Methods:** After obtaining the approval by the Ethics Committee of the Coordinating Center (IRCCS Ca’ Granda Ospedale Maggiore Policlinico, Milan), on March 2020 all the Italian birth centres were invited to participate. The database, designed by LP and CG and constructed on a REDCAP platform by CB, is composed of 5 sessions, asking for: birth hospital; mothers; infants born from a positive mother at the time of delivery; infants admitted for COVID infection acquired within the first month of life; infants born from a negative mother at delivery but positive during pregnancy. ES was responsible for data analysis.

**Results:** As of September 2020, 305 records were entered. Data analysis was performed on 291 infants, 238 of which born from a positive mother, 13 with an infection acquired after birth and 40 born from a mother with previous positivity. Most of the records were inserted by Lombardia (70.7%), Emilia Romagna (13%) and Piemonte (8.2%). Among the 278 infants born from a positive mother, 63% were delivered vaginally, 23% by elective and 14% by emergency CS. Of the 238 positive mothers at birth, 208 had a known positivity, 19 were under diagnostic assessment, and 11 showed symptoms and tested positive following delivery. At delivery, 23.5% of women were symptomatic, in most cases with mild to moderate flu-like symptoms. Fourteen% of infants had a GA<37wks showing a prematurity rate double than before the pandemic and in line with the literature data; 12.5% of infants had a BW<2500g. Seventy three% of infants born from positive mothers were isolated with her in rooming-in, 19.7% were isolated in the NICU, 2.9% in the Nursery, 2.5% with their mother and subsequently separated and 10.5% were transferred. Seventy nine% of infants were fed exclusively with breast milk. Overall, 2.8% of infants (n. 6) isolated in rooming-in with their mothers tested positive for SARS-CoV-2 during hospitalization. Of these, 1 was positive on day 1 and subsequently confirmed. Two were negative at birth and became positive during hospitalization, on day 7 and 9. Three were born to a mother not tested at delivery, but positive during hospitalization. In all cases, newborns were asymptomatic or paucisymptomatic. Thirteen infants were admitted for home-acquired SARS-CoV-2 infection; they were all symptomatic (mostly fever and feeding difficulties), requiring a ventilatory support only in 2 cases. The infection was contracted between 5 - 30 days of life and in 5 cases a contact with a positive family member was reported.

**Conclusion:** The COVID National Neonatal Registry improved epidemiological and scientific knowledge in this field and helped in creating a network which improves uniformity of management and high quality of care to infants and their mothers.

None of the authors have competing interest.

## A2: Feeding difficulties in the very preterm infant in the first year of life

### Eleonora Pontello ^1,2^, Federica De Osti ^1^, Nadia Battajon ^1^, Gianluca Visintin ^1^, Silvia Vendramin ^1^, Marika Buffo ^1^, Paola Lago ^1^

#### ^1^Neonatal Intensive Care Unit, Ca’ Foncello Hospital, Treviso, Italy; ^2^Department of Woman’s and Child’s Health, University of Padova, Padova, Italy

##### **Correspondence:** Eleonora Pontello (eleonora.pontello1@gmail.com)

**Background:** Feeding disorders affect about 25-45% of preschool children; they are especially common in children with developmental disorders, including premature babies^1^. They are one of the most common causes of delayed discharge, increased maternal stress and health care costs. During extrauterine life, the premature baby's nervous system is exposed to sensory overstimulation, resulting in an alteration of sensory processing and the highest risk of developing oro-buccal dyspraxia, hypersensitivity, and food aversion even after discharge ^2-20^. Several studies support an early intervention in NICU, in order to improve the maturation of infants’ proper oral feeding skills ^21-23^. Our primary outcome was to evaluate the incidence of feeding disorders on a VLBWI cohort during the 1st year of life. Secondary outcome was to identify any relationship between extreme prematurity, prolonged passive tube feeding and future feeding disorders.

**Materials and methods**: During NICU stay, we evaluated potential factors that could be correlated with the development of feeding difficulties, such as the duration of tube feeding, the transition time from tube to oral feeding, and the start of independent oral feeding. We examined also feeding difficulties in the first year of life and the incidence of GER, following in the weaning phase and at 12 months of life. We compare infant born GA≤28 with those ≥29 to understand the role of extreme prematurity in the development of feeding disorders. The demographic characteristics and clinical data were collected from electronic medical records by investigators. Prism 8 (GraphPad Software, San Diego, CA, USA) was used for statistical processing.

**Results**: We included 85 VLBW infants born in 2017-2018. The incidence of eating disorders in the first year of life was 29.4%. A statistically significant increase in feeding difficulties was found in VLBWIs with extreme prematurity (44.7% vs 17.0%, p <0.05), but there was no statistically significant difference in specific disorders (difficulty in weaning phase, difficulties at 12 months and GER).

**Conclusions:** Feeding disorders affect about one third of preterm babies in the first year of life. Poor feeding skills in infancy can continue to be problematic later on, for months or even years and become a serious concern for caregiver. This work helps to raise awareness among NICU staff, to reduce as much as possible the exposure to invasive procedures and to rehab these subjects early.

**KEYWORDS:** Feeding disorders; food aversion; sensory overstimulation; preterm; newborns.

**Patient permission**

Authors didn’t obtain written informed consent from parents for publication, since only anonymous unrecognizable patient data was used to fulfill the database.

**Acknowledgments**

We would like to thank all physicians and nurses of the Neonatal Intensive Care Unit at the Ca’ Foncello Hospital, Treviso, Italy, for support, advice, and commitment to providing high-quality care for these young patients and their families.

**References**

1. Lefton-Greif MA, Arvedson JC. Pediatric feeding and swallowing disorders: State of health, population trends, and application of the International Classification of Functioning, Disability, and Health. *Semin Speech Lang*. 2007;28(3):161-165.

2. Lau C. Development of infant oral feeding skills: What do we know? *Am J Clin Nutr*. 2016;103(2):616S-621S.

3. Lau C. Development of oral feeding skills in the preterm infant. *Handb Growth Growth Monit Heal Dis*. 2012;14:499-512.

4. Crapnell TL, Rogers CE, Neil JJ, Inder TE, Woodward LJ, Pineda RG. Factors associated with feeding difficulties in the very preterm infant. *Acta Paediatr Int J Paediatr*. 2013;102(12):539-545

5. Lau C, Smith EO. Interventions to improve the oral feeding performance of preterm infants. *Acta Paediatr Int J Paediatr*. 2012;101(7):269-274.

6. Thoyre SM. Feeding outcomes of extremely premature infants after neonatal care. *JOGNN - J Obstet Gynecol Neonatal Nurs*. 2007;36(4):366-376.

7. Muelbert M, Lin L, Bloomfield FH, Harding JE. Exposure to the smell and taste of milk to accelerate feeding in preterm infants. *Cochrane Database Syst Rev*. 2019;2019(7).

8. Griffith TT, Bell AF, White-Traut R, Medoff-Cooper B, Rankin K. Relationship Between Duration of Tube Feeding and Success of Oral Feeding in Preterm Infants. *JOGNN - J Obstet Gynecol Neonatal Nurs*. 2018;47(5):620-631.

9. Fowler C, Green J, Elliott D, Petty J, Whiting L. The forgotten mothers of extremely preterm babies: A qualitative study. *J Clin Nurs*. 2019;28(11-12):2124-2134.

10. Pagliaro CL, Bühler KEB, Ibidi SM, Limongi SCO. Dietary transition difficulties in preterm infants: Critical literature review. *J Pediatr (Rio J)*. 2016;92(1):7-14.

11. Hawdon JM, Beauregard N, Kennedy G. Identification of neonates at risk of developing feeding problems in infancy. Dev Med Child Neurol. 2000;42:235-9.

12. Rommel N, De Meyer AM, Feenstra L, Wausters GV. The complexity of feeding problems in 700 infants and young children presenting to a tertiary care institution. J Pediatr Gastroenterol Nutr. 2003;37:75-84.

13. Dodrill P, McMahon S, Ward E, Weir K, Donavan T, Riddle B. Long-term oral sensitivity and feeding skill of low-risk pre-term Infants. Early Hum Dev. 2004;76:23-37.

14. Törölä H, Lehtihalmes M, Yliherva A, Olsen P. Feeding skill milestones of preterm infants born with extremely low birth weight (ELBW). Infant Behav Dev. 2012;35:187-94.

15. Castro AG, Lima MC, Aquino RR, Eickmann SH. Desenvolvimento do sistema sensório motor oral e motor global em lactentes pré-termo. Pro Fono. 2007;19:29-38.

16. Mathisen B, Worral L, O’ Callaghan M, Wall C, Shepherd RW. Feeding problems and dysphagia in six-month-old extremely low birth weight infants. Adv Speech Lang Pathol. 2000;2:9-17.

17. Pridham K, Steward D, Thoyre S, Brown R, Brown L. Feeding skill performance in premature infants during the first year. Early Hum Dev. 2007;83:293-305.

18. Boer SL, Schipper JA. Feeding and drinking skills in preterm and low birth weight infants compared to full term infants at a corrected age of nine months. Early Hum Dev. 2013;89:445-7.

19. Demauro SB, Patel PR, Medoff-Cooper B, Posencheg M, Abbasi S. Postdischarge feeding patterns in early- and late-preterm infants. *Clin Pediatr (Phila)*. 2011;50(10):957-962.

20. Pineda R, Prince D, Reynolds J, Grabill M, Smith J. Preterm infant feeding performance at term equivalent age differs from that of full-term infants. *J Perinatol*. 2020;40(4):646-654.

21. Barlow SM, Finan DS, Lee J, Chu S. Synthetic orocutaneous stimulation entrains preterm infants with feeding difficulties to suck. J Perinatol. 2008;28(8):541-548.

22. Poore M, Zimmerman E, Barlow SM, Wang J, Gu F. Patterned orocutaneous therapy improves sucking and oral feeding in preterm infants. Acta Paediatr. 2008;97(7):920-927.

23. Greene Z, O'Donnell CP, Walshe M. Oral stimulation for promoting oral feeding in preterm infants. Cochrane Database Syst Rev. 2016;9(9):CD009720. Published 2016 Sep 20.

## A3: Neonatal early onset sepsis: impact of Kaiser calculator in an italian tertiary perinatal centre

### Eleonora Pontello ^1,2^, Nicoletta Mainini ^1^, Valentina Favero ^1^, Francesca Tormena ^1^, Michela Giovannini ^1^, Beatrice Galeazzo ^1^, Paola Lago ^1^

#### ^1^Neonatal Intensive Care Unit, Ca’ Foncello Hospital, Treviso, Italy; ^2^Department of Woman’s and Child’s Health, University of Padova, Italy

##### **Correspondence:** Eleonora Pontello (eleonora.pontello1@gmail.com)

**Abstract (word count 388)**

**Background:** A reappraisal of the guidelines for management of infants with early onset sepsis (EOS) advocates for close observation of well-appearing newborn infants ≥35 weeks’ gestation with maternal risk factors, rather than empiric antibiotic treatment ^1- 8^. EOS calculator represents a useful and safe tool to guide an individualized management of EOS, without any significant increases in adverse outcomes ^9-16^. Data on its clinical application is still limited. Our primary outcome was the rate of empiric antibiotics for suspected EOS in the first 72 hours. Secondary outcomes included the frequency of close or intensive monitoring of vital parameters, the rate of blood withdraw, the number of antibiotic use days per 100 live births.

**Methods:** We included newborn infants born at ≥35 weeks’ gestation at Treviso hospital in 2018 and 2019, with maternal EOS risk factors or EOS clinical signs. We compared 3 periods, before and after the introduction of EOS calculator, including a learning period and a fully application period. We described the effect of this calculator on clinical practice. The demographic characteristics and clinical data were collected from electronic medical records by investigators. Prism 8 (GraphPad Software, San Diego, CA, USA) was used for statistical processing.

**Results:** The study cohort included 4354 newborn infants with GA ≥35 weeks, respectively 826 in baseline period, 1426 in the learning period and 2102 in the EOS calculator period. Among them 1040 (23,8%) infants presented maternal risk factors for neonatal sepsis, including 216 (26,15%) in baseline period, 164 (11,5%) in learning period and 660 (31,3%) in EOS calculator period. Characteristics of the infants born in these 3 periods were similar for sex, gestational age, birth weight and delivery method. The incidence of culture-confirmed EOS was very low across 3 periods. Empirical antibiotic administration in the first 72 hours decreased respectively from 13,4% to 6,4% (p< 0,05). Blood culture and laboratory evaluations had fallen from 30,6% to 12,9% (p< 0,05). Close monitoring of vital parameters decreased from 99,1% to 13,8% (p< 0,05). The number of antibiotic days per 100 live births decreased from 17,07 to 8,94 days (p<0,05). We had no readmissions for EOS.

**Conclusions:** Application of EOS calculator is useful to standardize clinical practice as well as to reduce the use of antibiotics without compromising safety in a population with a relatively low incidence of culture-proven EOS and good access to follow-up care.

**Keywords:** Antibiotics; early onset sepsis; infection; newborns; sepsis calculator

**Patient permission**

Authors didn’t obtain written informed consent from parents for publication, since only anonymous unrecognizable patient data was used to fulfill the database.

**Acknowledgments**

We would like to thank all physicians and nurses of the Neonatal Intensive Care Unit at the Ca’ Foncello Hospital, Treviso, Italy, for support, advice, and commitment to providing high-quality care for these young patients and their families.

**References**

1. Kuhn P, Dheu C, Bolender C, Chognot D, Keller L, Demil H, et al. Incidence and distribution of pathogens in early-onset neonatal sepsis in the era of antenatal antibiotics. Paediatr Perinat Epidemiol. 2010;24(5):479–87.

2. Puopolo KM, Benitz WE, Zaoutis TE. Management of Neonates Born at ≥35 0/7 Weeks’ Gestation With Suspected or Proven Early-Onset Bacterial Sepsis. Pediatrics. 2018 Dec 1;142(6):e20182894.

3. Berardi A, Fornaciari S, Rossi C, Patianna V, Bacchi Reggiani ML, Ferrari F, et al. Safety of physical examination alone for managing well-appearing neonates ≥35 weeks gestation at risk for early-onset sepsis. J Matern Neonatal Med. 2015;28(10):1123–7.

4. Verani JR, McGee L, Schrag SJ. Morbidity and Mortality Weekly Report Prevention of Perinatal Group B Streptococcal Disease. Revised guidleines from CDC, 2010. Morb Mortal Wkly Rep. 2010;59(RR-10):1–36.

5. Centers for Disease Control and Prevention. 2018. Active Bacterial Core Surveillance Report, Emerging Infections Program Network, Group B Streptococcus, 2018. Available via the internet: http://www.cdc.gov/abcs/reports- findings/survreports/gbs18.pdf. Accessed on 01.03.2020.

6. Mukhopadhyay S, Taylor JA, Von Kohorn I, Flaherman V, Burgos AE, Phillipi CA, et al. Variation in sepsis evaluation across a national network of nurseries. Pediatrics. 2017;139(3).

7. Good PI, Hooven TA. Evaluating Newborns at Risk for Early-Onset Sepsis. Pediatr Clin North Am [Internet]. 2019;66(2):321–31.

8. Arora V, Strunk D, Furqan SH, Schweig L, Lefaiver C, George J, Prazad P. Optimizing antibiotic use for early onset sepsis: A tertiary NICU experience. J Neonatal Perinatal Med. 2019;12(3):301-312

9. Kuzniewicz MW, Puopolo KM, Fischer A, Walsh EM, Li S, Newman TB, et al. A quantitative, risk-based approach to the management of neonatal early-onset sepsis. JAMA Pediatr. 2017;171(4):365–71.

10. Achten NB, Klingenberg C, Benitz WE, Stocker M, Schlapbach LJ, Giannoni E, et al. Association of Use of the Neonatal Early-Onset Sepsis Calculator with Reduction in Antibiotic Therapy and Safety: A Systematic Review and Meta-analysis. JAMA Pediatr. 2019;173(11):1032–40.

11. Achten NB, Visser DH, Tromp E, Groot W, van Goudoever JB, Plötz FB. Early onset sepsis calculator implementation is associated with reduced healthcare utilization and financial costs in late preterm and term newborns. Eur J Pediatr. 2020;179(5):727–34.

12. Frymoyer A, Joshi NS, Allan JM, Cohen RS, Aby JL, Kim JL, et al. Sustainability of a Clinical Examination-Based Approach for Ascertainment of Early-Onset Sepsis in Late Preterm and Term Neonates. J Pediatr. 2020;

13. Goel N, Shrestha S, Smith R, Mehta A, Ketty M, Muxworthy H, et al. Screening for early onset neonatal sepsis: NICE guidance-based practice versus projected application of the Kaiser Permanente sepsis risk calculator in the UK population. Arch Dis Child Fetal Neonatal Ed. 2020;105(2):F118–22.

14. Strunk T, Buchiboyina A, Sharp M, Nathan E, Doherty D, Patole S. Implementation of the Neonatal Sepsis Calculator in an Australian Tertiary Perinatal Centre. Neonatology. 2018;113(4):379–82.

15. Leonardi BM, Binder M, Griswold KJ, Yalcinkaya GF, Walsh MC. Utilization of a Neonatal Early-Onset Sepsis Calculator to Guide Initial Newborn Management. Pediatr Qual Saf. 2019;4(5):e214.

16. Fowler NT, Garcia M, Hankins C. Impact of Integrating a Neonatal Early-Onset Sepsis Risk Calculator into the Electronic Health Record. Pediatr Qual Saf. 2019;4(6):e235.

## A4: Multi drug resistance in NICU and the rationale use of antibiotics. Donor breast milk and infection control. The experience of Meyer Children’s University Hospital

### Klaus Peter Biermann (klauspeter.biermann@meyer.it)

#### Hospital Infection Control Committee, Meyer Children’s University Hospital, Florence, Italy

**Background**

The Human Milk Bank (HMB) at the Meyer Children’s University Hospital was established in 1971. It is the first HMB in Italy and leader of the Tuscan Regional Network of HMBs since 2010.

Since its establishment, the Meyer’s HMB has processed more than 80,000 litres of milk, donated by more than 12,000 women, and supplied to more than 16,500 children [1].

The HMBs, functionally linked to paediatric departments, collect, store and distributes donor breast milk on medical prescriptions.

**Methods**

Women who choose to become a donor agree to undergo a simple but necessary screening, similar to that carried out at blood transfusion centres. The aim of this practice is to identify any clinical conditions or behaviour of the donor that may be harmful to the children who receive the donated milk [2].

The HMB follows the recommendations of international scientific associations [3, 4] and European Union directives on food hygiene (HACCP) in order to provide a product that meets the highest possible safety and integrity requirements for biologically active components. The quality of the product is guaranteed by well-established procedures regarding donor screening, milk collection and storage methods, physical and bacteriological controls, pasteurisation and documentation of medical-administrative acts. Donated, pasteurized milk is mainly provided to children admitted to the Meyer’s (about 300 patients per year); 20-25% of the bank milk is required by other public and private healthcare facilities.

The use of fresh human milk is temporarily contraindicated for newborns < 32 weeks (completed) of gestational age and/or immunodeficient neonates (T-cell deficiency) in case of women who contracted a CMV infection before pregnancy.

Positivity for HIV, HBV, HCV, drug use and alcoholism are conditions that permanently contraindicate the use of donated breast milk. Women with an ongoing syphilis and tuberculosis are temporarily excluded as donors.

**Results**

The consequent adoption of the method described above ensured the total absence of infectious diseases caused by the use of donor breast milk.

**Conclusion**

The reason for investing significant resources in a HMB is summarised by the main advantages of using human milk [5]:

- low incidence of necrotizing enterocolitis
- reduced incidence of sepsis and other infections
- reduced incidence of bronchopulmonary dysplasia
- high food tolerance
- prevention of hypertension and insulin resistance.

**References**

1. Profeti C, Belli FF, Magi L, Martini C, Matera M, Novelli S, Merusi I, Manfredi A, Domenici R, Tognetti S, Giampaoli B, Gasparre O, Pacini F, Avelardi N. La Rete regionale delle Banche del Latte Umano Donato (ReBLUD). Solidarietà e altruismo alimentano la vita dei bambini più vulnerabili. We People (2017) 4, 27-31.

2. Puglia M, Berti E, Bosi C, Ingargiola A, Magi L, Martelli E, Pratesi S, Sigali E, Tomasini B, Gagliardi L, Rusconi F e il Gruppo TIN Toscane on-line. Uso del latte materno e del latte umano donato nei neonati gravi pretermine in Toscana: archivio terapie intensive neonatali (TIN) Toscana On-line. https://www.epicentro.iss.it/ben/2017/luglio-agosto/1 (visited 10/06/2020)

3. Ministero della Salute. Comitato Nazionale Multisettoriale per l’allattamento materno. Linee di indirizzo nazionale per l’organizzazione e la gestione delle banche del latte umano donato nell’ambito della protezione, promozione e sostegno dell’allattamento al seno. Conferenza Stato-Regioni Accordo 5 dicembre 2013, ai sensi dell’art. 4 del Decreto legislativo 28 agosto 1997, n. 281 GU n. 32, 8 febbraio 2014

4. Società Italiana di Neonatologia. Linee Guida per la costituzione ed organizzazione di una Banca del latte Umano Donato. Trento: 2007.

5. DeMarchis A, Israel-Ballard K, Amundson Mansen K, Engmann C. Establishing an integrated human milk banking approach to strengthen newborn care. J Perinatol. (2017) 37, 469–474

## A5: Management of the mother-newborn dyad in SARS-CoV-2 positive mothers: rooming-in and breastfeeding

### G. Mangili^1^, M. Saruggia^1^, C. Milicia^2^, S. Ferrari^1^, M. Maino^1^

#### ^1^ UOC Patologia Neonatale-TIN, ASST Papa Giovanni XXIII, Bergamo, Italia; ^2^ Dipartimento di Pediatria, UOC Patologia Neonatale-TIN, ASST Papa Giovanni XXIII, Bergamo, Italia

##### **Correspondence:** G. Mangili (gmangili@asst-pg23.it)

Background

The SARS-CoV-2 pandemic has heavily impacted the Italian public health system, highlighting the urgency of guidelines for the mother-newborn dyad management.

Droplets and close contact are known to be a common route of viral transmission ^[1]^, while little is known about other routes, including the transplacental one. Transplacental transmission of SARS-CoV-2 infection is possible during the last weeks of pregnancy, but this remains still controversial ^[2-3]^.

Methods

Unlike other institutions, the Italian Society of Neonatology (SIN) reviewed the current scientific knowledge and assured that the mother-newborn dyad to the extent possible ^[4]^. Breastfeeding is not considered a transmission vehicle, neither for SARS-CoV-2 nor for other known respiratory viral infections (WHO, 2020). On the contrary, it has been found to be vehicle of specific SARS-CoV-2 antibodies within a few days following the onset of the disease in the mother, possibly modulating the clinical expression of infants’ infection.

Results

Consequently, SIN’s indications ^[5]^, endorsed by the Union of European Neonatal & Perinatal Societies (UENPS) are:

- Allow rooming-in and breastfeeding in asymptomatic mothers (Table1) ^[6-7]^;
- Separate symptomatic mothers from their baby until they are able to take care of her/him it;
- Expressed breast milk when possible, unpasteurised, to not reduce its biological and immunological value ^[8]^;
- Arrange differentiated intra-hospital paths, dedicated isolated rooms and use Personal Protective Equipment (PPE);
- Hospitalize premature or sick newborns of SARS-CoV-2 positive mothers in NICU isolated area, assisted by PPE-geared personnel;
- In case of SARS-CoV- 2 positive mothers, adopt strict hygiene measures: wash hands frequently and wear a surgical mask during breastfeeds and intimate contact with the newborn;
- Guarantee isolated room, prohibiting visits;
- Extend neonatal hospitalisation for 5-7 days;
- Swab test for newborns of SARS CoV-2-positive mothers at birth and before discharge; follow-up and swab test at 20 and 30 days of life.

Conclusion

These guidelines are designed by SIN to provide a better management of SARS-COV-2 positive mothers and their babies under an appropriate infection control. It has been clinically documented that, if adequate hygiene measures are respected, the SARS-CoV-2 virus is not spread from mothers to babies, even during their intimate contact at breastfeeding. Nonetheless, further research is needed to better understand this infection in all its epidemiological and clinical aspects.

**Bibliography**

1. Chan JF, Yuan S, Kok KH, To KK, Chu H, Yang J, et al. A familial cluster of pneumonia associated with the 2019 novel corona-virus indicating person-to-person transmission: a study of a family cluster. Lancet 2020;395:514e23

2. Patanè L, Morotti D, Giunta MR, Sigismondi C, Piccoli MG, Frigerio L, Mangili G, Arosio M, Cornolti G. Vertical transmission of coronavirus disease 2019: severe acute respiratory syndrome coronavirus 2 RNA on the fetal side of the placenta in pregnancies with coronavirus disease 2019-positive mothers and neonates at birth. Am J Obstet Gynecol MFM. 2020 Aug;2(3):100145. doi: 10.1016/j.ajogmf.2020.100145. Epub 2020 May 18. PMID: 32427221; PMCID: PMC7233206.

3. Vivanti AJ, Vauloup-Fellous C, Prevot S, Zupan V, Suffee C, Do Cao J, Benachi A, De Luca D. Transplacental transmission of SARS-CoV-2 infection. Nat Commun. 2020 Jul 14;11(1):3572. doi: 10.1038/s41467-020-17436-6. PMID: 32665677; PMCID: PMC7360599.

4. Davanzo R, Moro G, Sandri F, Agosti M, Moretti C, Mosca F. Breastfeeding and Coronavirus Disease-2019. Ad interim indications of the Italian Society of Neonatology endorsed by the Union of European Neonatal & Perinatal Societies. Matern Child Nutr. 2020 Apr 3:e13010. doi: 10.1111/mcn.13010.

5. Giusti A, Zambri F, Marchetti F, Sampaolo L, Taruscio D, Salerno P, Chiantera A, Colacurci N, Davanzo R, Mosca F, Petrini F, Ramenghi L, Vicario M, Villani A, Viora E, Zanetto F, Donati S. *Indicazioni ad interim per gravidanza, parto, allattamento e cura dei piccolissimi 0-2 anni in risposta all’emergenza COVID-19. Versione 31 maggio 2020*. Roma: Istituto Superiore di Sanità; 2020 (Rapporto ISS COVID-19 n. 45/2020)

6. Wyckoff, A. S. (2020). Rooming-in, with precautions, now OK in revised AAP newborn guidance. *AAP News (American Academy of Pediatrics)*, July 22, 2020.

7. CDCb. https://www.cdc.gov/coronavirus/2019-ncov/hcp/care-for-breastfeeding-women.html

8. UNICEF-UK-BFI: 2020. Statement on infant feeding on neonatal units during the coronavirus (COVID-19) outbreak. Updated 2 April 2020. https://www.unicef.org.uk/babyfriendly/wpcontent/uploads/sites/2/2020/04/Unicef-UK-Baby-Friendly-Initiative-statement-on-infant-feeding-during-the-Covid-19-outbreak.pdf

**Table 1 (abstract A5).** Indications on mother-infant management in the perinatal period

State of the Mother	Performing the SARS-CoV-2 RNA-PCR test on throat swab in the mother	Performing the RNA-PCR test for SARS-CoV-2 on throat swab in the newborn	Isolation of the mother°	Management of the newborn during hospitalization°	Breastfeeding advice	Prevention measures on mother-child contagion §
Asymptomatic or paucisymptomatic mother, known to be SARS-CoV-2 positive	Already done	YES	YES, in the dedicated postnatal area	YES, in a rooming-in regime, but in an isolated area dedicated to the puerperium	YES	YES
Paucisymptomatic mother	YES	Only if maternal test positive	YES, in a dedicated and isolated area of the puerperium waiting for the result of the laboratory test	YES, in rooming-in, isolated and dedicated area of the puerperium until the result of the laboratory test	YES	YES
-SARS-CoV-2 under investigation						
Mom with respiratory infection symptoms (fever, cough, discharge)	YES or already in progress	Only if maternal test positive	YES, in the dedicated area of the puerperium waiting for the result of the laboratory test	Newborn isolated and separated from the mother, at least until the result of the laboratory test. He is welcomed in the dedicated area of Neonatology (if asymptomatic) or NICU (if with respiratory disease) with the possibility of isolation	NO; use of expressed milk. **^**	YES
- SARS-CoV-2 positive or under investigation					Pasteurization is not indicated	

**§** Room divider or tent, face mask for the mother when she is breastfeeding or in intimate contact with the newborn, careful hand washing, arrangement of the baby's cradle at a distance of 2 meters from the mother's head, suspension of visits from relatives and friends

**°**In addition, adequate protection measures by health personnel, according to ministerial indications

**^**The mother's fresh milk must be expressed with a manual or dedicated electric breast pump. The mother should always wash her hands before touching the bottles and all components of the breast pump, following the recommendations for proper washing of the breast pump after each use

## A6: Development of neonatal nurses’ advanced skills in the management of pressure injuries in critical newborn

### Barbara Fassino, Laura Plevani, Silvia Ferrario

#### Foundation IRCCS Ca’ Granda Ospedale Maggiore Policlinico, Neonatal Intensive Care Unit, Milan, Italy

##### **Correspondence:** Silvia Ferrario (silvia.ferrario@policlinico.mi.it)

**Background.** The awareness that hospitalized infants might be at high risk of developing pressure injuries has increased in the last years. This is due to immature skin, compromised perfusion, decreased mobility, altered neurological responsiveness, fluid retention and medical devices. ^[1,2]^

Pressure injuries can be classified using the National Pressure Injuries Advisory Panel staging system based on the depth and severity of tissue injury. They can be also divided into conventional (caused by pressure on a bony prominence) or device-related (caused by pressure on the tissues from a medical device). ^[3]^

**Materials and methods.** A systematic review was performed. The aims of the study were: 1) to investigate incidence and risk factors of pressure injuries in neonatal population and 2) to analyze the most frequent neonatal pressure injuries. Secondary, preventive and therapeutic strategies were analyzed.

**Results.** Studies show that the incidence of pressure injuries is very variable in infants admitted to the Neonatal Intensive Care Units. ^[4]^

Infants develop both conventional and device pressure injuries: conventional pressure injuries are often located at the occiput because of the large dimension of this area, while device-pressure injuries are frequently caused by non-invasive ventilation devices on infants’ noses, particularly by Nasal Continuous Positive Airway Pressure (Ncpap). ^[2,4,5]^

Infants with Ncpap lesions are younger, have a lower weight and a lower gestational age than those with occipital pressure injuries ^[1,2,5]^ who are usually intubated, deeply sedated, in the post-surgery period and very edematous. ^[6,7,8]^

Proper identification of at-risk infants and the implementation of preventive strategies are crucial to reduce the incidence of pressure injuries. ^[9]^

Neonatal nurses should use validate neonatal skin risk assessment scales and develop protocols for the standardization of skin inspection and care, nutritional management and pressure management through specific dressings or special support surfaces. All nursing staff should know the basic rules for pressure injuries prevention, the possible support surfaces and the available options for treatment. ^[7,9,10]^

When a pressure injury unfortunately occurs, it is necessary to use the proper dressing. Few products are approved in newborns’ care due to the risk of possible adverse reactions using adult treatments. ^[8]^

**Conclusions.** Pressure injuries are a nursing care quality indicator and represent a relatively frequent, potentially preventable and critical event. ^[9]^

Implementation of an effective pressure injury prevention and treatment strategies program based on available scientific evidence is needed to reduce the variability of care. The benefits of standardized care include early risk identification and increasing and improving adherence to evidence-based preventive interventions. ^[11]^

Conflicts of interest

The authors have no conflicts of interest to disclose.

**References**

1. García-Molina P, Balaguer-López E, García-Fernández FP, Ferrera-Fernández MLÁ, Blasco JM, Verdú J. Pressure ulcers’ incidence, preventive measures, and risk factors in neonatal intensive care and intermediate care units. Int Wound J. 2018 Aug;15(4):571-579

2. Visscher M, Taylor T. Pressure ulcers in the hospitalized neonate: rates and risk factors. Sci Rep. 2014 Dec 11;4:7429

3. http://www.npiap.com (Accessed 1 Oct 2020)

4. García-Molina P, Alfaro-López A, García-Rodríguez SM, Brotons-Payá C, Rodríguez-Dolz MC, Balaguer-López E. Neonatal pressure ulcers: prevention and Treatment. Res Rep Neonatol. 2017 Sept;7 29-39

5. Imbulana DI, Manley BJ, Dawson JA, Davis PG, Owen LS. Nasal injury in preterm infants receiving non-invasive respiratory support: a systematic review. Arch Dis Child Fetal Neonatal Ed. 2018 Jan;103(1):F29-F35

6. Kulik LA, Connor JA, Graham DA, Hickey PA. Pressure injury prevention for paediatric cardiac surgical patients using a nurse-driven standardized clinical assessment and management plan. Cardiol Young. 2018 Sep; 28(9):1151-1162

7. Kriesberg Lange CP, Little JM, Mohr L, Kato K. Reducing Pressure Injuries in a Pediatric Cardiac Care Unit: A Quality Improvement Project. J Wound Ostomy Continence Nurs. 2018 Nov/Dec; 45(6):497-502

8. Meszes A, Tálosi G, Máder K, Orvos H, Kemény L, Csoma ZR. Lesions requiring wound management in a central tertiary neonatal intensive care unit. World J Pediatr. 2017 Apr;13(2):165-172

9. Noonan C, Quigley S, Curley MA. Skin integrity in hospitalized infants and children: a prevalence survey. J Pediatr Nurs. 2006 Dec; 21(6):445-53

10. Carol Turnage-Carrier, Kathleen M McLane, Mary Ann Gregurich. Interface pressure comparison of healthy premature infants with various neonatal bed surfaces. Adv Neonatal Care. 2008 Jun;8(3):176-84

11. Peterson J, Adlard K, Walti BI, Hayakawa J, McClean E, Feidner SC. Clinical Nurse Specialist Collaboration to Recognize, Prevent, and Treat Pediatric Pressure Ulcers. Clin Nurse Spec. 2015 Sep-Oct;29(5):276-82

## A7: Nursing research in neonatal intensive care unit: where are we?

### Patrizio Sannino (patrizio.sannino@policlinico.mi.it)

#### Fondazione IRCCS Cà Granda Ospedale Maggiore Policlinico, Direzione Professioni Sanitarie, Milan, Italy

**Background**

The quality of nursing care derives from the development of the knowledge that this discipline ^1,2^ Research plays e central role. The goal of nursing research is to strengthen and broaden current knowledge regarding nursing in order to contribute to performance improvement. Research is critical to meet the challenging goal of the delivering quality results in collaboration with clients, their families/their loved ones ^1,2,3,4^ By analyzing the literature produced in a specific sector, it is possible to have a picture of the cultural evoluttion of a science and its theoretical elaboration. The priorities of nursing research, identified in the study by Wielenga et al. ^5^ on the European NICUs, confirm identified neonatal intensive care nursing research priority provide a roadmap for future collaborative research efforts. The top nursing research priorities identified in our study relate to prevention and reduction of pain, medication errors, end-of-life care, the needs of parents and family, implementing evidence into nursing practice and pain assessment.

The study aims to describe the publications’ to nursing care in the neonatal setting, published in Europe in journals indexed in the main database.

**Methods**

A bibliometric search was conducted in july 2020 selecting the studies published between 2010 and 2020in international journals were searched on databases biomedical: PUB MED, EMBASE, and CINAHL.Each study has been classified on the base of qualitative and quantitative parameters.

**Results**

97 publications have been included. Results show the studies’ focus, the top nursing research priorities identified in the publications relate to: breastfeeding, palliative care /ethics, developmental care, education/ training, organization, pain, procedures. The main destination of the publications were national journals. The quantitative approach is more developed. In Italy and mainly concern single-center studies ^6^ . Qualitative, experimental and second research studies are still limited ^7^.

**Conclusions**

The results suggest e development of Italian nursing research in the last decade. However, more reading and publications in international scientific journals should be encouraged.

**Key words:** nurse’s role, nursing care, neonatal nursing, neonatal, newborn, infant, premature infant .

References

1. Davide Ausili, Agnese Boldrin, Benedetta Salimbeni, Stefania Di Mauro. Le pubblicazioni degli infermieri italiani su riviste internazionali: uno studio bibliometrico. L’infermiere, 2017;54:4:e55-e61

2. La ricerca infermieristica: uno strumento per la qualità dell’assistenza di Carlo Turci, Giuliana D'Elpidio, Giuliana Evangelisti, Carol Zullo “Infermiere Oggi” 2014 pag.3-14

3. Metodologia della ricrca infermieristica. Lobiondo Wood GeriI; Haber JugithH; Palese A. (CURATORE) Editore: THE MCGRAW-HILL COMPANIES pag 6-10

4. Goldfrad C, Vella K, Bion JF, et al. Research priorities in critical care medicine in the UK. Intensive Care Med 2000;26:1480–8.

5. Joke M Wielenga , Lyvonne N Tume , Jos M Latour , Agnes van den Hoogen European Neonatal Intensive Care Nursing Research Priorities: An e-Delphi StudyArch Dis Child Fetal Neonatal Ed. 2015 Jan;100(1):F66-71.

6. Annamaria Bagnasco, Roger Watson, Michela Barisone, Ramona Pellegrini, Fiona Timmins, Giuseppe Aleo, Valentina Bressan, Lucia Cadorin, Nicoletta Dasso, Dario Valcarenghi,Gianluca Catania, Milko Zanini, Loredana Sasso. Lo sviluppo della ricerca infermieristica in Italia a dieci anni dall’istituzione delle scuole dottorali. Professioni Infermieristiche, Vol. 72 (3) Luglio – Settembre 2019

7. Valentina Bressan , Angela Tolotti , Michela Barisone , Annamaria Bagnasco, Loredana Sasso, Giuseppe Aleo, Fiona Timmins Perceived Barriers to the Professional Development of Modern Nursing in Italy - A Discussion Paper Nurse Educ Pract 2016 Mar;17:52-7

## A8: Self-scheduling of nurses’ shifts in NICU

### Mattia Luciano^1^, Paola Serafini^3^, Donato Mastrantuono^3^, Maria Chiara Ariotti^2^

#### ^1^NICU of AOU City of Health and Science of Turin, Turin, Italy; ^2^NICU of AOU City of Health and Science of Turin, Turin, Italy; ^3^Direction of health professions of AOU City of Health and Science of Turin, Turin, Italy

##### **Correspondence:** Mattia Luciano (mattia.luciano@unito.it)

**BACKGROUND**. The management of human resources represents a fundamental organizational variable of healthcare companies that is reflected transversally and significantly on the assistance provided. For the scheduling of the shifts of healthcare workers, basic and manual tools are used such as spreadsheets or simple sheets of paper that do not allow timely changes, with the risk of exposing themselves to non-coverage of the service, to trace the changes made over time and to manage access according to permissions for privacy levels. Proper shift planning should take into account the internal rules of the organization, the legal and contractual regulations, the skills required in each shift and the preferences of individual people [1]. In fact, offering self-programmed control to health personnel (i.e. the ability to decide the hours / days of work) is a feasible intervention as a solution to increase employee satisfaction [2]. The aim of this work was to design, build and implement an application (App) for scheduling and managing work shifts, oriented towards self-programming, for healthcare personnel.

**METHODS**. A multidisciplinary group was defined to create the App. It was composed by IT engineers/technicians, from the LINKS Foundation, and a representation of the health professions of the Obstetrics-Neonatal-Gynecological area of the Turin AOU Città della Salute e della Scienza. The App design and implementation regarded the limits analysis (regulatory, contractual and corporate); this was necessary to define the work shift and which method, in terms of application, could be more functional. Afterward, the study focused on the App validation, implementation and experimentation phase within the TIN-U department with sufficient knowledge in the use of the most common devices (PC, Tablet and Smartphone).

**RESULTS**. The IT tool, called “Mamma Roster” (MR), was designed and developed as a web application, in order to permit the use any device, both desktop and mobile. A web browser was defined/used to favor the internet and intranet using. Users were able to access to the tool after authentication, with username and password, and could have one or more roles with different privileges and functions. In particular: nurses and OSS (users) were able to program only the personal planning (working days, rest and absences) and request shifts changes from colleagues; the coordinator, on the other hand, was able to access to the complete planning of all users, manage everyone shifts, start the automatic generation of a schedule, and create new users. To ensure the functioning of the system, rules managed by the server have been identified and inserted, saved in the database, settled by the nursing coordinator via a settings page. The needs request was structured by establishing a degree of priority to avoid a binding system and a diffuse unhappiness among users. In addition, the actions permitted within the reserved area of ​​users were regulated by precise automatic timings to ensure adequate scheduling of shifts after entering of the needs.

**CONCLUSION**. In computer science, creating a schedule for the shifts of healthcare workers is a typical decision-making problem that belongs to the NP-hard class, a range issues that are often impossible to solve efficiently. Management science, operations research and information technology offer many solutions ranging from simple algorithms to more complex systems but do not always take into account the needs of operators. A shiftwork organization model, that considers the preferences of health personnel, improves their attitude, towards shift work, and alleviates work stress [3]. The use of the “Mamma Roster” software will determine further advantages, thanks to imposed rules and the possibility of automatic generation of planning.

**References**

1. Drouin, R, Potter M. Flexible scheduling. Amer. J. Nursing. 2005; 105(11) 72E–72F.

2. Robb EA, Determan AC, Lampat LR, ScherbringMJ, Slifka RM, Smith NA. Self- scheduling: Satisfaction guaranteed. Nursing Management. 2003; 34(7) 16–18.

3. Ha EH. Attitudes towards rotating shift work in clinical nurses: a Q- methodology study. J Clin Nurs. 2015 Sep;24(17-18):2488-97.

## A9: Bonding and its importance in mother infant relationship

### Assunta Coppola, Anna Pentangelo, Roberto Cinelli

#### Operative Unit of Neonatology and Neonatal Intensive Care. Presidio Ospedaliero San Leonardo, Castellammare di Stabia, Asl Na3Sud

##### **Correspondence:** Assunta Coppola (susycoppola@hotmail.it)

**Background**

Neonatal bonding is that particular bond between mother and her newborn that begins in the mother's womb and is consolidated at birth immediately after childbirth. It is an important practice, which has a significant influence on the psychological component of the newborn. Indeed, it plays an essential role in supporting early neonatal social interactions, which can influence the neuro-behavioral outcomes of late childhood. Bonding consists of different elements that interact with each other: skin to skin contact, early and exclusive breastfeeding, eye contact and rooming-in.

**Methods**

The aim of this study is to assess glucose stability, breastfeeding rates at discharge and nutritional needs comparing groups of women who gave birth vaginally and by caesarean section. For this purpose, the responses of a group of 224 women were examined. We a randomly selected sample of women who had given birth from September 2019 and February 2020 in maternity and neonatology ward of the San Leonardo Hospital of Castellammare di Stabia, which has about 1000 births a year with a strong prevalence of vaginal births compared to caesarean ones. The sample included mothers of healthy and full-term infants. The women had an average age of 31 years with a range from 16 to 47 years old. In particular, 80,36% of women gave birth vaginally, 14,29% planned caesarean section and 5.35% by emergency caesarean birth.

Statistical analysis was conducted by means of the Chi-square tests to estimate, with 95% confidence intervals, differences between categorical variables.

**Results and conclusions**

This work showed the correlation between the practice of bonding and the absence of glycemic changes in infants born from vaginal birth compared to those born from caesarean section, for which bonding is not performed. No differences between the two types of birth were found, with a confidence interval of 95%, both in terms of breastfeeding and the nutritional needs of each newborn.

## A10: Undetected congenital heart disease: old and new tools to recognize them

### Iuri Corsini (corsiniiuri@gmail.com)

#### Division of Neonatology, Careggi University Hospital, Largo Brambila 3, 50134, Florence Italy

**Background:** Congenital heart disease (CHD) represent an important cause of morbidity and mortality in the neonatal period but also in later ages. [1]⁠ Prenatal diagnosis remains a very important tool in the diagnosis of these pathologies. Literature shows that only 50% of CHD is recognized during fetal life, [2]⁠ with wide variability from center to center and depending on the type of CHD. Neonatologist therefore plays a fundamental role in the diagnosis and management of these conditions.

**Discussion:** Congenital heart defects can occur with acute clinical onset, immediately after birth or in the first hours or days of life. In these cases the cardiopathies involved are generally those characterized by non-duct dependent mixing circulation (i.e. total pulmonary anomalous venous return or transposition of the great arteries) or by ductus dependencies of the systemic or pulmonary circulation. These heart diseases generally manifest themselves clinically with cyanosis or shock. The neonatologist can use various tools to reach the diagnosis such as: clinical history, physical examination (finding of murmurs, reduction or absence of femoral pulses, etc.), hyperoxia test, etc. However in an emergency setting echocardiography remains essential. The use of this tool by the neonatologist has become widespread in recent years, as shown by recent literature. [3, 4]⁠ Recently, European scientific societies have also drawn up guidelines in an attempt to standardize the use of this method. [5]⁠

Certain types of CHD could manifest themselves later in life, even after months or years from birth. For these conditions, proactive research by the pediatrician who follows the child is essential. Thus oximetry screening has entered in current clinical practice. [6]⁠ However, this instrument has some limitations such as the low detection rate for CHD characterized by left ventricle outflow obstructive defects (i.e. aortic coarctation, aortic stenosis, hypoplastic left heart, etc.). [7]⁠

In later ages, CHD can manifest with variable symptoms such as growth retardation, poor exercise tolerance, electrocardiographic modifications.

**Conclusion:** It is important for the pediatrician to always remember, at any age, that the child may be suffering from a congenital heart disease. The proactive research of these diseases, using all available tools, will ensure the best possible care for children.

**Acknowledgment**

The author would like to thank all board members ﻿of the study group of neonatal cardiology of the Italian society of neonatology for their collaboration and support: Dr Irma Capolupo, Dr Rosa Maria Cerbo, Dr Daniela Doni, Dr Benjamim Ficial, Dr Stefano Fiocchi, Dr Gaia Francescato, Dr Federico Matina, Dr Fabio Mizzoni, Dr Sabrina Salvadori, Dr Marilena Savoia.

**References**

1. Wren C, Richmond S, Donaldson L (1999) Presentation of congenital heart disease in infancy: implications for routine examination. Arch Dis Child - Fetal Neonatal Ed 80:F49–F53. DOI: 10.1136/fn.80.1.F49

2. Brown KL (2006) Delayed diagnosis of congenital heart disease worsens preoperative condition and outcome of surgery in neonates. Heart 92:1298–1302. DOI: 10.1136/hrt.2005.078097

3. Corsini I, Ficial B, Fiocchi S, et al (2019) Neonatologist performed echocardiography (NPE) in Italian neonatal intensive care units: a national survey. Ital J Pediatr 45:131. DOI: 10.1186/s13052-019-0721-z

4. Savoia M, Porzio S (2020) Legal issues in Neonatologist Performed Echocardiography. The Italian experience. Pediatr Res 87:1140–1142. DOI: 10.1038/s41390-019-0721-0

5. de Boode WP, Singh Y, Gupta S, et al (2016) Recommendations for neonatologist performed echocardiography in Europe: Consensus Statement endorsed by European Society for Paediatric Research (ESPR) and European Society for Neonatology (ESN). Pediatr Res 80:465–471. DOI: 10.1038/pr.2016.126

6. Martin GR, Ewer AK, Gaviglio A, et al (2020) Updated Strategies for Pulse Oximetry Screening for Critical Congenital Heart Disease. Pediatrics 146:e20191650. DOI: 10.1542/peds.2019-1650

7. Schena F, Picciolli I, Agosti M, et al (2017) Perfusion Index and Pulse Oximetry Screening for Congenital Heart Defects. J Pediatr 183:74-79.e1. DOI: 10.1016/j.jpeds.2016.12.076

## A11: Light and shade in the physiology and treatment of neonatal low cardiac output syndrome

### Savina Mannarino^1^, Irene Raso^2^, Luisa Federica Nespoli^1^, Carla Giuseppina Corti^1^

#### ^1^Pediatric Cardiology, Department of Pediatrics, University of Milan, Children's Hospital V Buzzi, Milan, Italy; ^2^Cardiology, Department of Cardiology, University of Pavia, IRCCS Policlinico San Matteo, Pavia, Italy

##### **Correspondence:** Irene Raso (irene.raso2407@gmail.com)

The imbalance between oxygen delivery (D02) and consumption (V02) leads to morbidity, mortality and adverse neurodevelopmental outcomes in premature infants.Central and peripheral factors define the amount of oxygen available to cells. Haemoglobin concentration, arterial oxygen partial pressure and, above all, cardiac index and output (CO) are the central factors, while microcirculation, haemoglobin affinity and CO redistribution are the peripheral factors.

In the prematurenewborn, the cardiovascular system is immature and frequently leads to low-systemic blood flow (LSBF) states (35% of <30-week-old and61% of <27-week-old-infants). The low cardiac output syndrome (LCOS) is defined as a condition caused by a transient decrease in the systemic perfusion withVO2/DO2 imbalance at the cellular level. Immature organs are vulnerable to hypoxic damage and a differential diagnosis between LSBF and LCOS is crucial. The transitional circulation makes the CO monitoring particularly difficult. Treating low mean arterial pressure without signs of organ hypoperfusion represents an oversimplification of the physiopathology and can beevena damaging intervention. The neonatologists should adopt all the diagnostic tools (eco-functional, Non-Invasive CO Monitor, Near-infrared spectroscopy) to try to detect inadequate tissue perfusion and oxygenation at an early stage.

In the suspicion of LCOS, clinicians should think to the possible contributing factors and the clinical context and should consider adaptation/maladaptation to a dynamic cardiocirculatory change. Contributing factors include reduced preload, reduced contractility, increased afterload and reduced vascular resistances. Moreover, it is important to consider the clinical context, the prenatal conditions and the time of LCOS onset.

The management of LCOS include drugs that can be categorized as predominantly vasopressors (dopamine, norepinephrine) or inotropes (dobutamine, milrinone, levosimendan). Epinephrine is an inotropic drug with dose-dependent vasoactive action. The choice of the therapeutic agent should accurately follow the pathophysiology of the disease, considering the action on the heart and the peripheral receptor profile, with specific attention to the effect on systemic/pulmonary flows and resistances.

In conclusion, newborns with hemodynamic compromise require careful approach, due to the peculiarity of their neonatal circulation, the immaturity of the organism and the different responses to stimuli. Further studies are required to improve the monitoring of patients and the understanding of the individual response to inotropic agents.

